# Transactions of the Mississippi State Medical Association

**Published:** 1877-03-20

**Authors:** 


TRANSACTIONS OF THE MISSISSIPPI STATE MEDICAL ASSOCIATION.—We are indebted to our friend and co-laborer, Dr. Robert Kells, of Jackson, Miss., for a copy of the transactions of the State Medical Association of Mississippi. We have not space for a detailed notice of the many valuable reports and papers contained in this neatly executed and valuable compilation. It speaks well for the intelligence and enterprise of the Physicians of Mississppi. Its contents are—*The President's Address*, M. W. S. Croft; *Electro-Therapeutics*, by Dr. R. G. Wharton; *Ramollisment*, by Dr. Compton; *Cerebro-Spinal Meningitis*, by Dr. Browning; *Hemipligia Kittrell—Antiseptic Treatment of Wounds*, and a case of *Necrosis*, &c., by Dr. J. M. Taylor; *Capillary Bronchitis* in children, by Dr. R. Fowler, *Chloroform and Ether*, by Dr. E. Banks; *Indiginous Remedies*, by Dr. B. H. Whitfield: *Topography and Diseases* of North Mississippi, by Dr. E. W. Hughes; *Memorial Notices*, by Drs. Coffman and Petrie.

